# Size- and polymer-dependent toxicity of amorphous environmentally relevant micro- and nanoplastics in human bronchial epithelial cells

**DOI:** 10.1186/s43591-025-00126-9

**Published:** 2025-05-16

**Authors:** I. F. Gosselink, P. Leonhardt, E. M. Höppener, R. Smelt, M. J. Drittij, M. Davigo, G. G. H. van den Akker, I. M. Kooter, T. J. M. Welting, F. J. van Schooten, A. H. V. Remels

**Affiliations:** 1 Department of Pharmacology and Toxicology, Institute of Nutrition and Translational Research in Metabolism (NUTRIM), Maastricht University, Universiteitssingel 50, 6629 ER Maastricht, the Netherlands; 2 Netherlands Organisation for Applied Scientific Research, TNO, 3584 CB Utrecht, the Netherlands; 3 Laboratory of Experimental Orthopedics, Department of Orthopedic Surgery, Maastricht University, 6229 ER Maastricht, the Netherlands

**Keywords:** Inhalation toxicology, Polypropylene, Polyamide, Polyvinylchloride, Inflammation, NF-ĸB

## Abstract

**Background:**

Knowledge of the toxicological impact of micro- and nanoplastics (MNPs) on the human airway epithelium is limited and almost exclusively based on experiments applying high doses of spherical polystyrene (PS) particles. In this study, we investigated the toxicity of a broad size range of amorphous MNPs generated from different environmentally-relevant polymers.

**Methods:**

Bronchial epithelial cells (BEAS-2B) were exposed to three different doses of polyvinylchloride (PVC), polypropylene (PP), or polyamide (PA) particles (< 1 μm-10 μm), as well as leachates from these polymers. Toxicity was evaluated by assessment of cytotoxicity, inflammation (IL-8 release and inflammatory gene expression) and oxidative stress (DCFH-DA assay and antioxidant gene expression). Furthermore, the molecular mechanism behind MNP-induced inflammation was investigated by studying activation of two well-known inflammation related transcriptional factors (NF-κB and AP-1).

**Results:**

Only PA nanoplastics induced significant cell death, IL-8 secretion and inflammatory gene expression compared to vehicle control. PA-induced inflammation was accompanied by NF-κB, but not AP-1, transcriptional activity. PA did not increase cellular ROS levels; however, it did lead to increased expression of the antioxidant gene superoxide dismutase 2. In addition to PA, exposure to < 1 µm and 1–5 µm PP particles resulted in elevated IL-8 secretion, likely due to the presence of talc added as filler. None of the leachates affected cytotoxicity or inflammation.

**Conclusion:**

Toxicity of MNPs to human bronchial epithelial cells was dependent on polymer type, size and dose. Nanoplastics, especially PA, were more toxic to bronchial epithelial cells than microplastics and induced cytotoxicity and an inflammatory response.

**Graphical Abstract:**

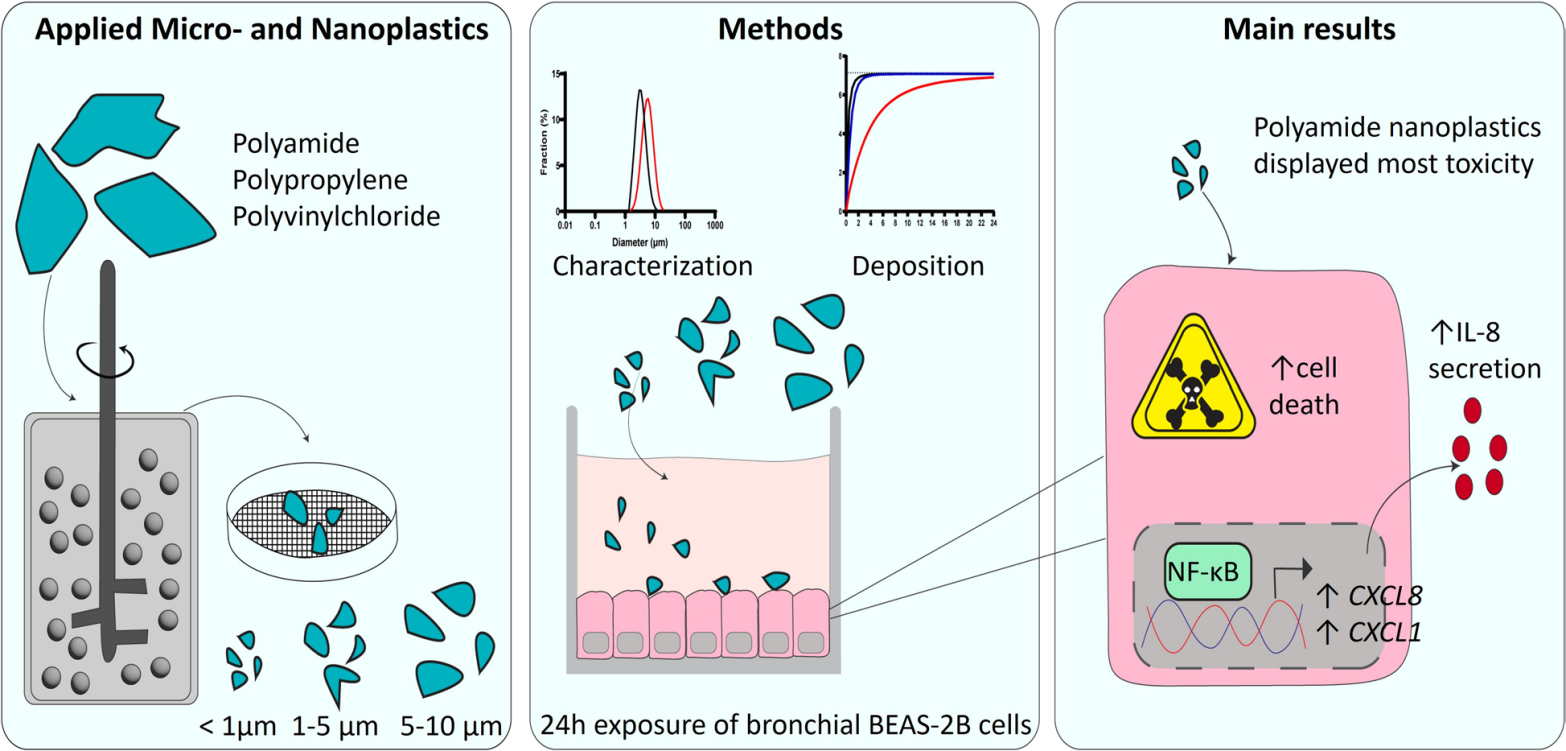

**Supplementary Information:**

The online version contains supplementary material available at 10.1186/s43591-025-00126-9.

## Introduction

Humans are constantly exposed to microplastics (< 5 mm) and nanoplastics (< 1 μm) (MNPs) [1]. Whereas the ingestion exposure route has received quite some scientific attention, less is known about the impact of inhalation of MNPs. Atmospheric MNPs can originate from diverse sources including landfills, synthetic textiles, erosion of synthetic rubber tires and industrial emissions [2, 3]. Dris et al. [4] were the first to measure atmospheric fallout of microplastics in a case study conducted in the Greater Paris area, observing an average of 118 particles/m^2^/day with a higher concentration detected in indoor environments than outdoors. Histopathological findings of MNPs in human lung tissue have confirmed exposure via inhalation [5]. Furthermore, correlations between lung diseases and exposure to high amounts of airborne MNPs have been reported in workers in the plastic industry [6, 7]. This all poses significant concerns about the interaction between MNPs and human respiratory health. However, the extent of potential toxic effects of MNPs in bronchial epithelial cells and underlying molecular mechanisms remain to be identified.

Cytotoxic effects of polystyrene (PS) have been observed in bronchial BEAS-2B epithelial cells [8, 9]. Additional mechanistic studies have identified the activation of apoptotic pathways as an underlying mechanism of PS-induced cytotoxicity [10]. Also, in the same cells, PS exposure has been associated with increased IL-6 and IL-8 secretion as well as elevated reactive oxygen species (ROS) levels, indicating the induction of inflammation and oxidative stress as important pathways for MNP-associated toxicity [11]. However, most studies addressing toxicity of MNP solely focused on applying high doses of spherically shaped PS particles. These particles have a uniform morphology and chemical composition and therefore do not reflect the diversity of airborne MNPs humans are exposed to [12, 13].

With regard to which polymers can be considered as environmentally relevant, indoor air analysis has identified the presence of microplastics derived from various polymers including polyamide (PA), polyethylene terephthalate (PET), polypropylene (PP) and polyvinyl chloride (PVC) [14, 15]. In addition, a recent study identified 12 different polymer types in human lung tissue, with PP (23%) and PET (18%) being the most prevalent, while PS particles accounted for only 8% of total MNPs detected [5]. In addition to polymer type, surface chemistry, shape and size are additional key parameters influencing toxicity [8, 16, 17]. Indeed, smaller MNPs penetrate deeper into the human lung and have a higher surface area to volume ratio [18, 19]. Since an increased surface area might highly influence MNP-cell interactions, we hypothesized a size-dependent effect of MNPs on (cyto)toxicity. In addition to particle-induced toxicity, MNPs can release chemicals used in the production process of plastics (so-called leachates), which could potentially induce chemical toxicity [20]. In support of this notion, leachates from PA fibers have recently been shown to negatively affect growth and differentiation of both developing airway organoids and air–liquid interface cultures of primary bronchial epithelial cells [21].

Research on the potential toxic effects of environmentally relevant airborne MNPs is very limited. Therefore, in this study, we aimed to investigate the potential toxicity of environmentally relevant MNPs of PP, PA, or PVC, as well as their associated leachates, in human bronchial epithelial cells (BEAS-2B cells). MNPs in three different size distributions, ranging from < 1 µm to 10 µm, were produced via a standardized milling procedure by the MOMENTUM research consortium on Microplastics and Human Health [22]. Leachates were collected during the MNP synthesis and were tested in different concentrations in our cell system. Talc, which was used as a filler for PP (to decrease buoyancy), was included as well. To provide insights into the impact of MNPs on human bronchial epithelial cells, toxicological end points included cytotoxicity, inflammation and oxidative stress.

## Materials and methods

### Materials and reagents

Dulbecco’s Modified Eagle Medium (DMEM, 61,965–026), Hank’s Balanced Salt Solution (HBSS), Dulbecco’s Phosphate-Buffered Saline (dPBS), Fetal bovine serum (FBS), Penicillin–Streptomycin (P/S), and Trypsin–EDTA (0.25%), as well as Recombinant TNF-α, TRIzol reagent, and GlycoBlue coprecipitant were purchased from Thermo Fisher Scientific (Waltham, USA). The following chemicals were purchased from Sigma-Aldrich (St. Louis, USA): Triton X-100, Chloroform, TWEEN® 20, Bovine Serum Albumin (BSA), dichlorofluorescein diacetate (DCFH-DA), puromycin, glutathione (GSH), Glutathione disulfide (GSSG), monopotassium phosphate (KH_2_PO), Potassium phosphate dibasic trihydrate (KH_2_PO · 3H_2_O), 5-sulfosalicyclic acid (SSA), 5,5-dithiobis(2-nitro-benzoic acid) (DTNB), Nicotinamide Adenine Dinucleotide Phosphate reduced tetrasodium salt (NADPH), Glutathion reductase, 2-Vinylpyridine (VP), EthyleneDiamineTetraaceticAcid DiSodium Salt (Na-EDTA), Quercetin and absolute Ethanol. The IL-8/CXCL8 ELISA kit, as well as Substrate Reagent and Stop Solution for ELISA assays were obtained from R&D Systems (Minneapolis, USA). The Cytotoxicity Detection Kit was supplied from Roche (Basel, Switzerland). The iSCript™ cDNA synthesis kit was purchased from Bio-Rad (Hercules, USA), and the SensiMixTM SYBR® & Fluorescein Kit from Bioline (London, UK). The Nano-Glo® reagent was provided from Promega (Madison, USA) and IKK-16 was purchased from Selleck Chemicals LLC (Houston, USA).

Environmentally relevant MNPs of PVC, PP mixed with Talc (PP/Talc) and Polyamide 6.6 (PA) were produced by centrifugal and ball-milling and supplied in suspension in 1-propanol by the MOMENTUM consortium [22, 23]. Three different size fractions were included in our study: < 1 µm, 1–5 µm, and 5–10 µm. The production and fractioning protocol is described in detail by Parker et al*.* [22]. Talc was added to PP as a filler during the production process to increase the density and prevent buoyancy of the particles. Talc, in milled and non-milled form was also provided in 1-propanol suspension.

### Preparation of particle suspensions

Working solutions were prepared directly before exposure. All particles were first coated in 0.5% Bovine Serum Albumin (BSA) to increase steric repulsion and prevent agglomeration, and then further diluted to desired particle concentration while reaching a final concentration of 0.05% BSA, 1% Fetal bovine serum (FBS), and 1% Penicillin–Streptomycin (P/S) in Dulbecco’s Modified Eagle Medium (DMEM). As MNPs stock suspensions were synthesized in 1-propanol, final concentrations of 1-propanol were kept equal for all conditions at 1% of the total volume.

### Characterization of suspensions

#### Particle morphology

Scanning electron microscopy (SEM) was used on the particles to confirm the size and shape of the MNPs. The suspended particles were filtered under reduced pressure (~ 200 mbar) over 25 mm gold or nickel coated polycarbonate filters (TJ Environmental, Hilversum, the Netherlands) with pore sizes 0.1 µm (smallest fractions of PVC and PP/Talc) or 0.8 µm (all other particles) (TJ Environmental, Hilversum, the Netherlands). The filter was transferred onto an aluminium stub with a carbon coated tape. Using a Q150 T carbon evaporator the samples were coated with a thin layer of carbon to ensure an electronically conductive surface for SEM analysis. (Quorum, Laughton, UK) SEM measurements were performed with a MAIA III GMH field emission scanning electron microscope (Tescan, Brno, Czech Republic). SEM images were recorded using a secondary electron (SE) detector and operated with acceleration voltages between 5–15 kV.

#### Particle size distribution

Suspensions of PVC, PA, PP/Talc MNPs and talc were characterised using Static Light Scattering (SLS) on a LA-960 particle size analyser (HORIBA, Kyoto, Japan) with a 405 nm laser. Working solutions were prepared as described above (paragraph 'Preparation of particle suspension') and measured at 100 μg/mL in 0.05% BSA, 1% FBS, 1% P/S in DMEM under constant movement at 23 °C, ~ 50% relative humidity. Refractive indices were set to 1.55–0.00I for PVC, and 1.50–0.00I for PA, 1.60–0.00I for PP/Talc, and 1.60–0.00I for talc respectively. Using the LA-960 software (HORIBA), an average spectrum from five measurements was generated and the average median diameter was calculated (volume based; D50_V_).

#### Zeta-potential

The zeta-potential of 100 μg/mL suspensions in 1-propanol (PP/Talc, PVC and PA) was determined by using a Zetasizer nano ZS (Malvern Panalytical), after equilibration in the instrument for 120 s.

#### Effective density

The effective density for all MNP particles and talc in exposure medium has been determined using the volumetric centrifugation method. The principles of this method has been extensively described in DeLoid et al. [24].

First, particle suspensions were incubated overnight at 37 °C. Thereafter, a pellet was obtained by benchtop centrifugation of the suspensions in a packed cell volume tube (Sigma-Aldrich). The volume of the pellet was observed by two independent observers using an easy read device (TPP Techno Plastic Products AG, Switzerland). The pellet volume (V_pellet_), in combination with the media density (ρ_media_) applied mass (M_ENM_) and bulk polymer density (ρ_ENM_) was used to calculate the effective density using the equation below. The stacking factor (SF) for the particles was presumed to be 0.634 (random close stacking).

$${\rho }_{\text{EV}}={\rho }_{\text{media}}+\left[\left(\frac{{M}_{\text{ENM}}}{{V}_{\text{pellet}}SF}\right)\left(1-\frac{{\rho }_{\text{media}}}{{\rho }_{\text{ENM}}}\right)\right].$$

#### Solvent parameters

Media density was calculated from the average of five measurements of the mass of a 1 mL sample by subtracting the weight of an empty vial from the weight of the vial containing 1 ml of exposure media at 37 °C. Viscosity of the exposure medium was measured five times with an ubbelohde Viscometer, type 501 01.0a.

### BEAS-2B cell culture and exposure

Human bronchial epithelial cells, BEAS-2B, were obtained from American Type Culture Collection (ATCC) and maintained in DMEM supplemented with 10% FBS and 1% P/S (full medium) in a humidified 5% carbon dioxide atmosphere at 37° C. Full medium was replaced approximately every two days and cells were passaged before confluency. 24 h prior to exposure, cells were seeded in 48-wells plates with a seeding density of 30,000 cells/well.

BEAS-2B cells were exposed in 400 μL particle suspensions (paragraph 'Preparation of particle suspensions') to three different MNPs concentrations: 2.2 µg/mL, 6.6 µg/mL, or 19.6 µg/mL for 24 h. Since PP/Talc MNPs consisted of 26–42% talc [22], tested talc concentrations were 0.7 µg/mL, 2 µg/mL, or 5.9 µg/mL, corresponding with 30% of the PP/Talc dose. A vehicle control, 1% FBS, 1% 1-propanol, 0.05% BSA, was included in all experiments.

In addition, we exposed cells to leachates of PVC, PP/Talc, and PA particles that were collected during the production process. Here, leachate stock solutions (in 1-propanol), were diluted 100x, 300× or 1000x. Vehicle controls for the leachates contained the corresponding amount of 1-propanol in those experiments (0.11%, 0.33%, or 1%).

TNF-α (50 ng/mL) exposure for 24 h served as a positive control for inflammation in each experiment. In all experiments, exposure was done in three technical replicates, and each experiment was performed a minimum of three times (biological replicates).

To study the effect of inhibiting the Nuclear factor kappa-light-chain enhancer of activated B cells (NF-κB) pathway, cells were incubated with IKK-16 (0–0.3 µM) 45 min before and during PA-exposure. DMSO concentrations were 0.001% in all conditions.

In experiments with anti-oxidants, cells were seeded 48 h before PA-exposure. 24 h before exposure, cells were pre-incubated with Quercetin (10–40 µM) or full medium, each containing a final DMSO concentration of 0.05%. At the moment of exposure, medium was replaced by exposure medium containing PA with/without Quercetin (10–40 µM), again with a final DMSO concentration of 0.05%.

### Dosimetry

After 24 h exposure to particles, cells were imaged using a DM IL LED inverted microscope (Leica, Wetzlar, Germany) at 10× magnification, as an indicator for particle behavior in medium. Furthermore, the deposition of the MNPs was modelled using the RiskGONE in vitro dosimetry web application, which is part of the Horizon 2020 RiskGONE project and based on the Harvard DG model developed by Deloid et al*.* [25, 26]. The following parameters were used in the model: polymer density, as provided by the manufacturer of the bulk plastic (Table 1); effective density as measured via volumetric centrifugation (paragraph ‘Characterization of suspensions’); solvent parameters (paragraph ‘Characterization of suspensions’), suspension column height = 3.64 mm, height of sub-compartment = 0.02, centrifugation = 1 (gravity), total simulation time = 24 h, time interval for simulation = 1 s.

Size distribution data of MNPs incubated for 24 h in exposure medium are obtained via SLS (paragraph ‘characterization of suspensions’) and uploaded as fraction distribution by volume. No further dissolution was assumed. In the end, the estimated deposited dose of the particles was reported in units of particle mass per unit surface area (µg/cm^2^), which additionally was transformed to number of particles per cm^2^ (Table S2).

### Cytotoxicity assay

Following 24 h exposure, supernatant was collected for Lactate dehydrogenase (LDH) analysis to determine cytotoxicity according to manufacturer’s specifications (Cytotoxicity Detection Kit (LDH) Roche AG). Absorbance was measured at 492 nm with a SpectraMax iD3 Multi-Mode Microplate reader (Molecular Devices, San Jose, USA). These values were corrected by both a wavelength correction (650 nm) and medium background. To reach maximum cytotoxicity (LDH max), cells were exposed to Triton-X-100/DMEM (2%) for 10 min. Cytotoxicity was expressed as percentage of LDH max.

### IL-8 ELISA

The IL-8/CXCL8 ELISA Duoset kit was used to quantify IL-8 protein secretion after exposure, according to the manufacturer’s protocol (R&D Systems). Absorbance measurements were performed at 470 nm using the SpectraMax iD3 Multi-Mode Microplate reader. Measurements were corrected for a background measurement (570 nm) and a blank control. IL-8 protein levels were calculated based on the standard curve, using a four-parameter logistic regression curve.

### Real-time quantitative-PCR

Total RNA from exposed BEAS-2B cultures was extracted using TRIzol reagent following the Invitrogen User Guide for TRIzol RNA isolation. GlycoBlue was added as co-precipitant. RNA concentrations and quality was determined using the microvolume spectrophotometer NanoDrop One (Thermo Fisher Scientific). 400 ng of RNA was converted to cDNA using the iScript™ cDNA synthesis kit, according to the manufacturer’s instructions. A no enzyme control (NEC), as well as a no template control (NTC) were included. Quantitative polymerase chain reaction (qPCR) was performed using the SensiMix™ SYBR® & Fluorescein Kit. Each reaction contained 1.76 ng cDNA and primers (0.3 µM) specific for genes of interest in white LightCycler480 384 multiwell plates (BRAND GMBH + CO KG, Wertheim, Germany) (Table S1). The following thermal cycling protocol was performed using the LightCycler® 480 (Roche, Basel, Switzerland): 10 min at 95 °C, 55 cycles of 10 s at 95 °C, 20 s at 60 °C. Quantitative gene expression analysis was performed in LightCycler480 software 1.5.1 (Roche) and LinRegPCR software 2014. The geometric mean of four housekeeping genes (beta-actin *(ACTB)*, beta-2 microglobulin (*B2M*), Cyclophilin A (*CYPA*), and ribosomal protein L13 A (*RPL13 A*)) was used for normalisation of gene expression (Table S1).

### DCFH-DA assay

The DCFH-DA assay was used to measure the levels of ROS within the cells. BEAS-2B cells were seeded 24 h before exposure, in a 96-well plate at a seeding density of 2.5 × 10^5^ cells/well. First, cells were incubated for 40 min with 10 µM DCF-DA in DMEM without phenol red. Thereafter, cells were exposed to either 1000 µM H_2_O_2_ (positive control) or PA nanoplastics in phenol red-free exposure medium. Phenol red-free exposure medium was used as vehicle control. The fluorescent DCF signal was measured after 1 h, 2 h, 3 h and 24 h at 485 nm excitation and 535 nm emission wavelengths using a spectramax ID3 reader.

### Measurement of GSH and GSSG levels

BEAS-2B cells were seeded 24 h before exposure in a 6-well plate. After 24 h exposure to either PA nanoplastics or vehicle, cells were lysed in Potassium phosphate lysis buffer (KPE) containing 5 mM EDTA and 1% Triton-X, whereafter the supernatant was mixed with SSA (final concentration SSA 1.3%). Glutathione disulfide/glutathione (GSSG/GSH) levels were determined with a GSH-assay, based on a publication of Rahman et al*.* [27]. First, standards of both GSH (0.1–10 μM) and GSSG (0.1–5 μM) were prepared in KPE buffer. To measure GSSG, both GSSG standards and samples were incubated for 1 h with VP, to allow the VP to form a stable complex with GSH. To measure total GSH, samples were not treated with VP. After incubation, reactions were initiated by adding 0.4 mM NADPH/0.3 mM DTNB and 4 U/ml GSSG reductase to the samples. Absorbance of the samples and standards was recorded kinetically for 5 min in 10 reads at 415 nm at 37 °C using a spectramax ID3 reader, resulting in GSH + GSSG and GSH slope values. Data are presented as GSH:GSSG ratios.

### NF-κB and AP-1 transcriptional activity assay

To generate BEAS-2B cells that report of the activity of NF-κB and Activator protein-1 (AP-1) transcription factors, we transduced BEAS-2B cells with lentivirus carrying Nano Luciferase driven by NF-κB (RelA/p50) or AP-1 (Fos/Jun) transcription factor binding elements, as described earlier [28]. Stable BEAS-2B reporter cells were obtained by selecting transduced cells using 2 μg/ml puromycin. Following puromycin selection cells were used in luciferase reporter experiments with MNPs. Transduced BEAS-2B cells were seeded 24 h before exposure, in a 96-well plate. After 24 h exposure to either PA nanoplastics or vehicle cells were washed with PBS, lysed in MQ and subsequently the Nano-Glo® reagent was then added to each well in 1:1 with milliQ water with a Tristar 2 injection system (Berthold Technologies, Bad Wildbad, Germany). Luminescence was quantified using the Tristar2 LB942 multi-mode plate reader (Berthold Technologies).

### Statistical analysis

Data obtained were analyzed on mean values from each biological replicate with the statistical software GraphPad Prism version 10.3.1 (GraphPad Software, San Diego, USA). Assumptions for normality and homogeneity of variance were checked using a Shapiro–Wilk test and Brown-Forsythe test respectively. Statistical differences between vehicle and treatment conditions were tested using an ordinary one-way ANOVA with Dunnett’s multiple comparisons test (normally distributed data) or a Kruskal–Wallis test (non-normally distributed data). For TNF-α, a two-tailed unpaired t test was used to test statistical differences. For all tests, significance was assumed at *p* < 0.05.

## Results

### Characterization of MNP suspensions

**Fig. 1:**
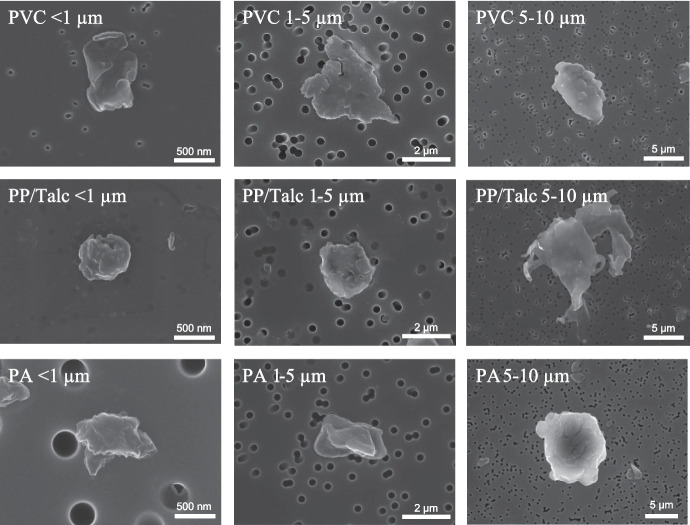
Particle shape characterization. Representative Scanning electron microscopy (SEM) images of the different size fractions of Polyvinylchloride (PVC), Polypropylene/Talc (PP/Talc) and Polyamide (PA) micro- and nanoplastics

**Fig. 2:**
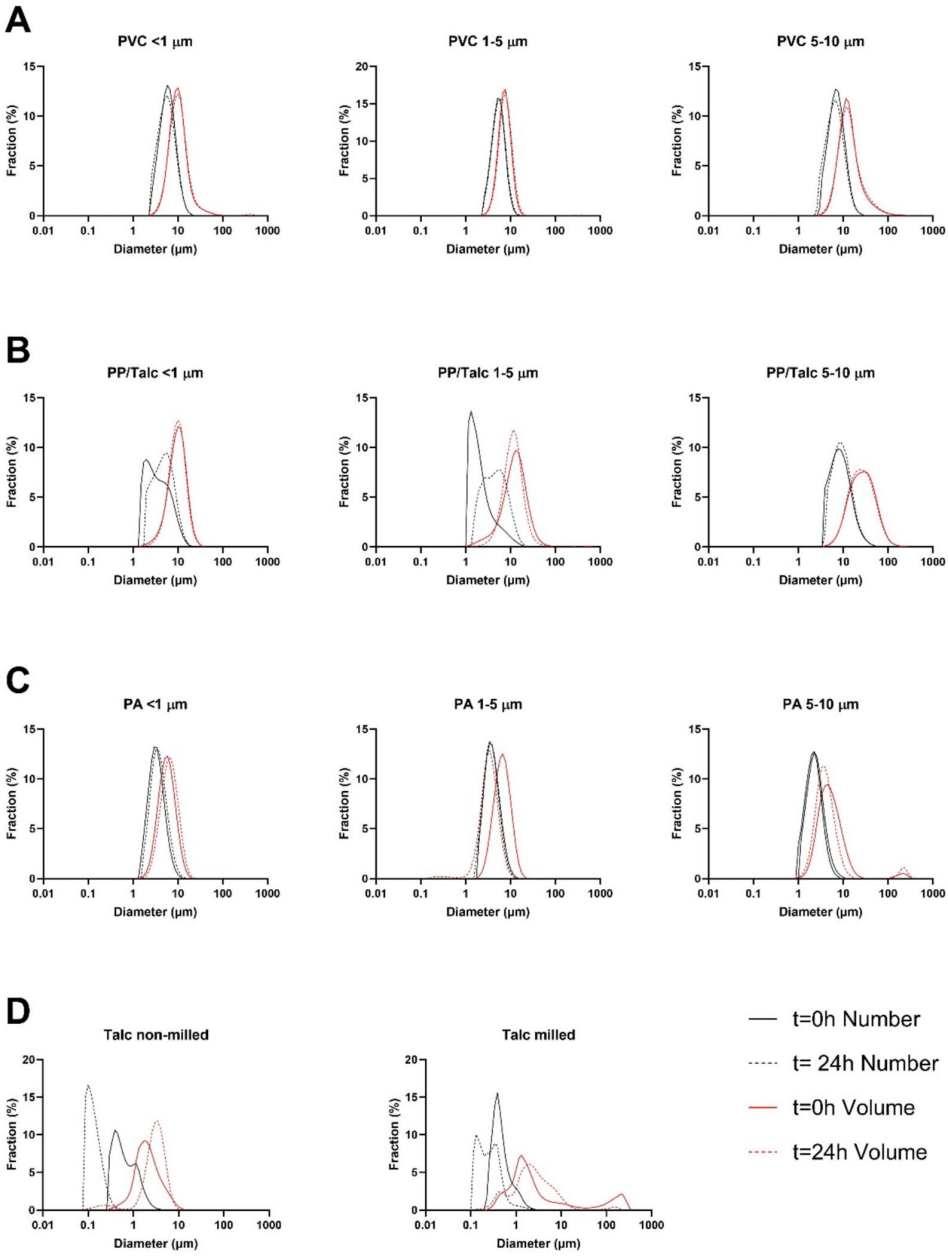
Static Light Scattering (SLS) measurements of micro- and nanoplastics (MNPs) and talc. Representative number-based (black lines) and volume-based (red lines) distribution spectra are shown for **A** polyvinylchloride (PVC), **B** polypropylene/talc (PP/Talc), **C** polyamide (PA) and **D** talc. MNPs and talc incubated for 0 h and 24 h in exposure medium (DMEM, 0.05% BSA, 1% FBS, 1% 1-propanol)

Particles used in this study were extensively characterized in terms of morphology, size distribution, and surface charge. Representative SEM images indicate that the MNPs have an irregular shape, and the fragments were all variable in dimensions and shape (Fig. 1). Whereas the two smallest size fractions displayed a round morphology, PP/Talc MNPs of fraction 5–10 µm appeared to have a more irregular shape, with protrusions from the edges. Particle size distributions (PSD) were characterized with SLS. MNP suspensions of PVC, PP, and PA had a broad size distribution. The median number-based size (D_N_, Table 1) of all fractions in 1-propanol were similar to the primary particle diameters as shown in the SEM images (Fig. 1). The Zeta potential value for the MNP suspensions ranged from − 5.5 mV to − 17.3 mV in 1-propanol (Table 1). After dispersion in exposure medium, an increase in D_N_ was observed for the smallest size fraction (< 1 µm) for particles of all polymers, indicating agglomeration (Table 1). The median volume-based size, D_v_, normally considered to be dominated by larger particles, is bigger than the primary particle size for suspensions in propanol and exposure medium. The MOMENTUM MNPs displayed a heterogeneous size in exposure medium, as can be observed from broader SLS size distribution peaks (Fig. 2). Milled talc displayed a broader size distribution and increase in median size compared to non-milled talc, both in 1-propanol as in exposure medium (Fig. 2), indicating agglomeration of the milled talc [23]. The size distribution for MNPs did not change during the time of exposure (24 h), with the exception of the PP/Talc particles in the two smallest fractions that showed a small increase in (number-based) median size (Fig. 2). Also for talc, an increase of (volume-based) median size was detected, although non-agglomerated particles are still present as can be observed from the number-based size distributions. In the presence of cells, agglomeration of particles was observed in brightfield pictures taken after 24 h exposure (Fig. S1).

**Table 1:** Physicochemical characteristics of particles. The mean Zeta potential (in mV) ± SD was measured for Polyvinylchloride (PVC), Polypropylene/Talc (PP/Talc), Polyamide (PA) and talc. The median diameter (D_N_ number basis and D_V_ volume basis) was measured in 1-propanol and exposure medium (DMEM, 1% FBS, 0.05%BSA, 1% 1-propanol), using static light scattering. The effective density was determined via volumetric centrifugation of the suspensions. The polymer density was provided by the manufacturer. *Size distributions in 1-propanol are derived from earlier publications [22, 23]

	**Zeta potential (mV)**	**D**_**N**_**,** **D**_**V**_** (μm)** in 1-propanol*	**D50**_**N**_**,** **D50**_**V**_** (μm)** **in exposure medium**	**Effective density (g/cm**^**3**^**)**	**Polymer density (g/cm**^**3**^**)**
**PVC < 1 μm**	−5.5 ± 0.8	0.21 5.72	5.45 9.17	1.00	1.38
**PVC 1–5 μm**	−13.6 ± 0.2	1.47 4.65	4.96 6.83	1.16	1.38
**PVC 5–10 μm**	−17.3 ± 1.23	4.17 10.61	6.79 11.82	1.13	1.38
**PP/Talc < 1 μm**	−11.9 ± 0.7	0.40 5.83	3.15 9.16	1.08	1.25
**PP/Talc 1–5 μm**	−8.8 ± 0.8	4.28 6.36	1.73 11.74	1.02	1.12
**PP/Talc 5–10 μm**	−8.3 ± 1.6	10.57 24.16	7.89 24.54	1.01	1.12
**PA < 1 μm**	−9.5 ± 4.2	0.68 5.60	3.05 5.23	1.02	1.14
**PA 1–5 μm**	−8.1 ± 0.6	1.42 3.98	3.55 6.05	1.01	1.14
**PA 5–10 μm**	−9.3 ± 0.8	2.11 2.31	2.20 4.53	1.05	1.14
**Talc milled**	−6.7 ± 0.1	0.81 1.21	0.40 1.63	1.09	2.75
**Talc non-milled**	−8.4 ± 0.2	0.65 1.10	0.54 1.83	0.98	2.75

Chemical characterization of PP, PA and PVC MNPs, as determined by X-ray Fluorescence by a previous study, established that the total chemical contamination was < 1 wt% [22, 23]. Detected elements included low traces of phosphorus, iron, chromium, nickel and zirconium, most likely introduced during the ball-milling of the particles in vessels from stainless steel.

### Delivered dose computation

To accurately determine the amount of MNPs that reached the cells in our experiments, the delivered dose was estimated according to the RISKGone in vitro dosimetry model. This model is based on the Harvard distorted grid model developed by Deloid et al*.* [25, 26]. Input parameters consider sedimentation and diffusion velocities of the particles in exposure medium. When particles are dispersed in medium, their density is often lower compared to the polymer density of the raw material due to agglomeration, interaction with proteins and trapped media fluid. This effective density influences the particle’s sedimentation rate and was therefore obtained for all MNPs as well as for the milled and non-milled talc. Indeed, all particles showed an effective density lower than the polymer density (Table 1). In addition to polymer and effective particle density, the RISKGone in vitro dosimetry model inputs included the size distribution by volume, as determined by SLS (D_v_ t = 24 h) and solvent parameters, such as the media density (0.98 g/cm^3^) and media dynamic viscosity (0.730 mPa.s). Applied concentrations for MNPs during the experiment were 2.2 µg/mL, 6.6 µg/mL and 19.6 µg/mL for the low, medium and high dose respectively. The maximum dose, in case of 100% deposition efficiency would correspond with 0.79 µg/cm^2^, 2.38 µg/cm^2^, and 7.14 µg/cm^2^. The estimated deposition over time after applying 19.6 µg/mL MNPs is displayed per particle in Fig. S2. Although the estimated deposition of PVC nanoplastics (6.90 µg/cm^2^) was similar to the deposition of PVC plastics of the two bigger fractions (7.10 µg/cm^2^), it took longer for nanoplastics before total deposition was reached. For PP/Talc, the estimated deposition over time was similar for all particles, ultimately leading to ± 7.00 µg/cm^2^ total particle deposition for all fractions (Table S2). Whereas the predicted deposited dose over 24 h for PVC and PP/Talc nearly reached 100% of the applied dose, the estimated fraction of PA particles that deposited was lower. The estimated dose was 6.80 µg/cm^2^, 4.22 µg/cm^2^ and 6.38 µg/cm^2^ for PA < 1 µm, PA 1–5 µm and PA 5–10 µm respectively (Table S2). Furthermore, the estimated deposition of milled talc reached 64% of the applied dose. For the non-milled talc deposition was estimated to be low, and could not be calculated with the in vitro dosimetry model, due to the low effective density of this particle.

### PA nanoplastics induce cytotoxicity

**Fig. 3:**
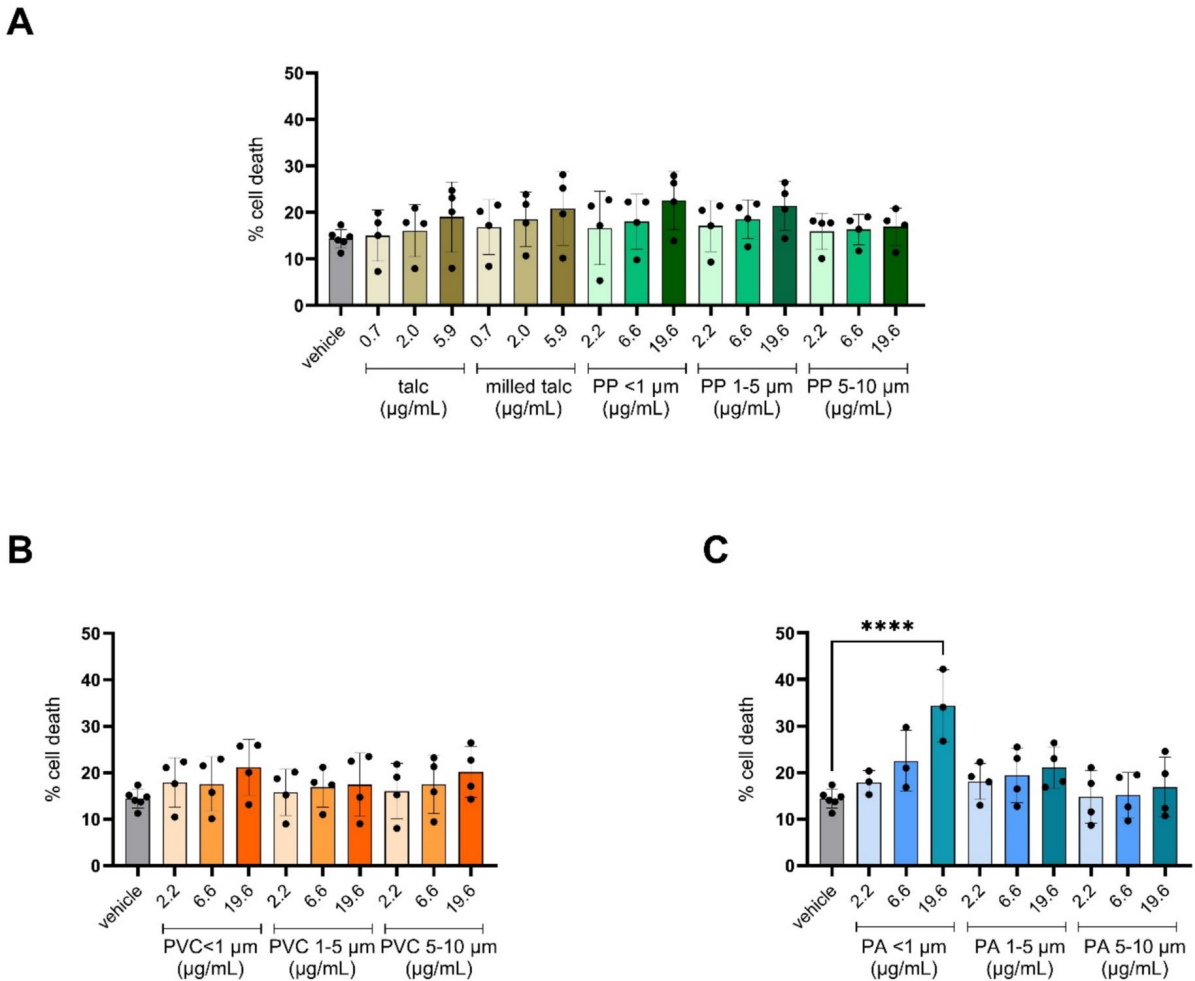
Evaluation of cytotoxicity upon exposure to polypropylene mixed with talc (PP/Talc), polyvinyl chloride (PVC), and polyamide (PA). Cells were exposed to the vehicle control (exposure medium; DMEM, 0.05% BSA, 1% FBS, 1% 1-propanol) or to MNPs of the respective polymers in exposure medium. Applied doses are displayed in the bar charts, in μg/mL. Released lactate dehydrogenase (LDH) levels were measured after 24 h exposure. Maximum LDH release was achieved by exposure to Triton X-100 (2%). Cytotoxicity data upon exposure to PP/Talc and talc (**A**), PVC (**B**), and PA (**C**) MNPs in size fractions of < 1 µm, 1–5 µm and 5–10 µm. Every data point represents an independent exposure experiment, each performed in technical triplicate. Data presented as mean percentage of the maximum LDH release ± SD. ****P < 0.0001 compared to vehicle control

Cytotoxicity upon 24 h exposure to PP/Talc, PVC or PA particles of different size fractions (< 1 µm, 1–5 µm or 5–10 µm) was assessed by determining the LDH release in the supernatant. We observed no induction of cytotoxicity in response to PP/Talc MNPs of all sizes nor the adjuvant talc and milled talc (Fig. 3A). Similarly, no significant increases in cytotoxicity were observed upon exposure of the cells to PVC particles (Fig. 3B). On the other hand, exposure to PA nanoplastics (< 1 µm size fraction) did induce significant cytotoxicity in a dose-dependent manner (Fig. 3C). The larger size fractions PA (1–5 µm and 5–10 µm) did not induce cytotoxicity (Fig. 3C). To distinguish whether the cytotoxic effects observed were due to physical effects of the MNPs or to the chemicals that were transferred, we exposed cells to leachates (LTs) of each particle at concentrations of 0.11%, 0.33% and 1% LT solution. No changes in cell death were observed for any of the leachates (Fig. S3A).

### Pro-inflammatory effects in response to PA nanoplastics and adjuvant talc

**Fig. 4:**
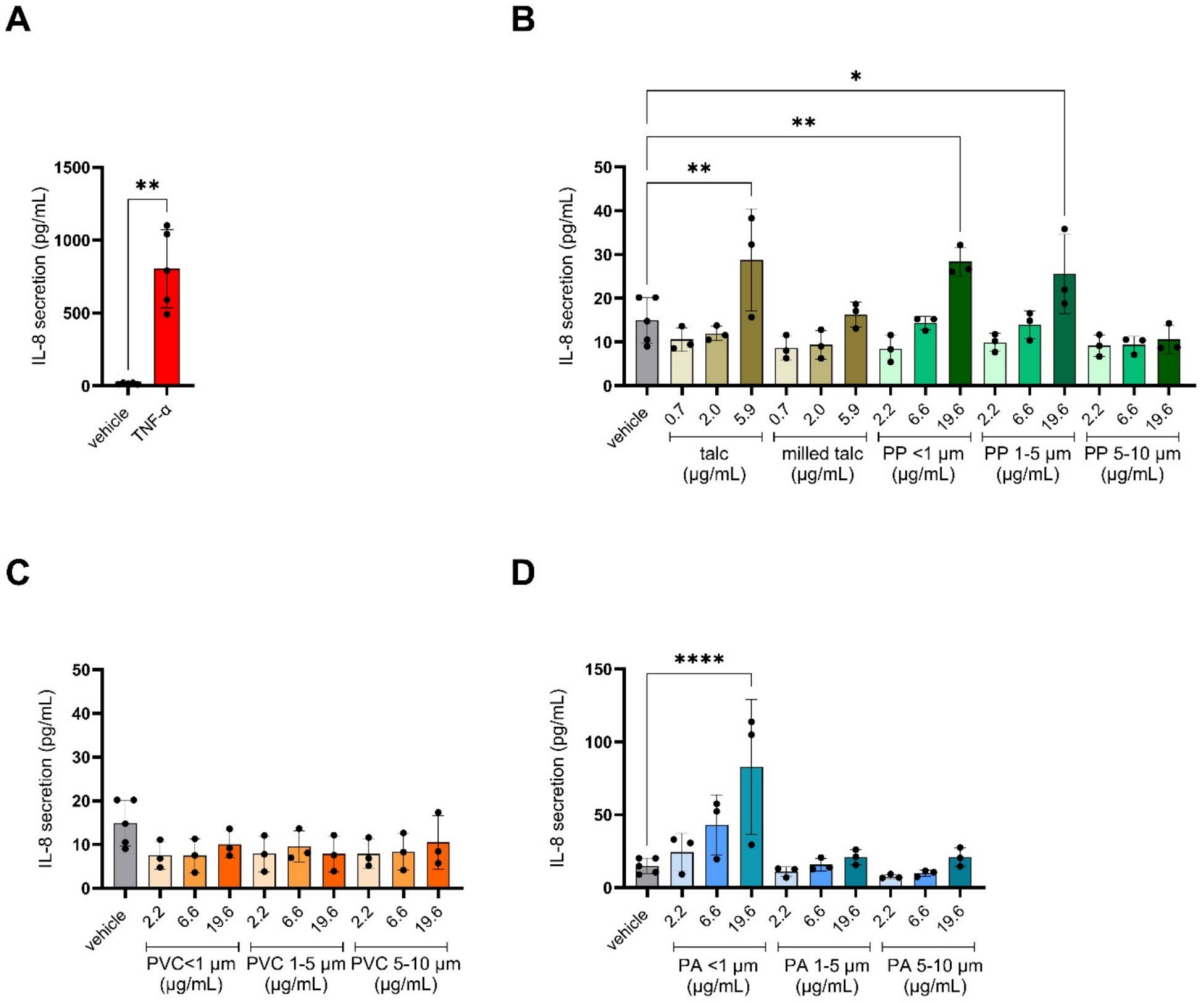
IL-8 secretion upon exposure to polypropylene mixed with talc (PP/Talc), polyvinyl chloride (PVC), and polyamide (PA) micro- and nanoplastics. Cells were exposed to the vehicle control (DMEM, 0.05% BSA, 1% FBS) or to the different size fractions of the respective polymers. Applied doses are displayed in μg/mL. IL-8 secretion upon parallel exposure to positive control TNF-α (50 ng/ml) (**A**), PP/Talc (**B**), PVC (**C**) and PA (**D**) in size fractions of < 1 µm, 1–5 µm and 5–10 µm. Talc and milled talc doses were identical to quantities of talc present in the PP/Talc particle suspensions. Every data point represents an independent exposure experiment, each performed in technical triplicate. Average absolute IL-8 levels (± SD) are compared with the vehicle control: *P < 0.05; **P < 0.01; ****P < 0.0001

After assessing cytotoxicity, the inflammatory potential of the MNPs, their respective leachates as well as talc was assessed by measuring IL-8 secretion in the medium. IL-8 is well-described to be produced in response to various stimuli including pathogens, cigarette smoke and pollutants, and plays a significant role in acute lung injury as a potent chemoattractant for neutrophils [29]. TNF-ɑ, included as positive control, potently induced IL-8 secretion compared to the vehicle control (Fig. 4A). With regard to the MNPs and their leachates, none of the leachates induced IL8 secretion in any of the concentrations tested (Fig. S3B). In contrast, the highest dose of PP/Talc particles of < 1 µm, as well as 1–5 µm, increased IL-8 secretion compared to vehicle control as did talc in its non-milled form (Fig. 4B). No difference in IL-8 secretion was observed upon exposure to PVC particles (Fig. 4C). IL-8 secretion increased significantly upon exposure to the PA nanoplastics (< 1 µm) in a dose-dependent manner. Larger PA MNPs (1–5 µm and 5–10 µm) did not induce significant amounts of IL-8 secretion (Fig. 4D).

### PA-induced inflammatory processes

**Fig. 5:**
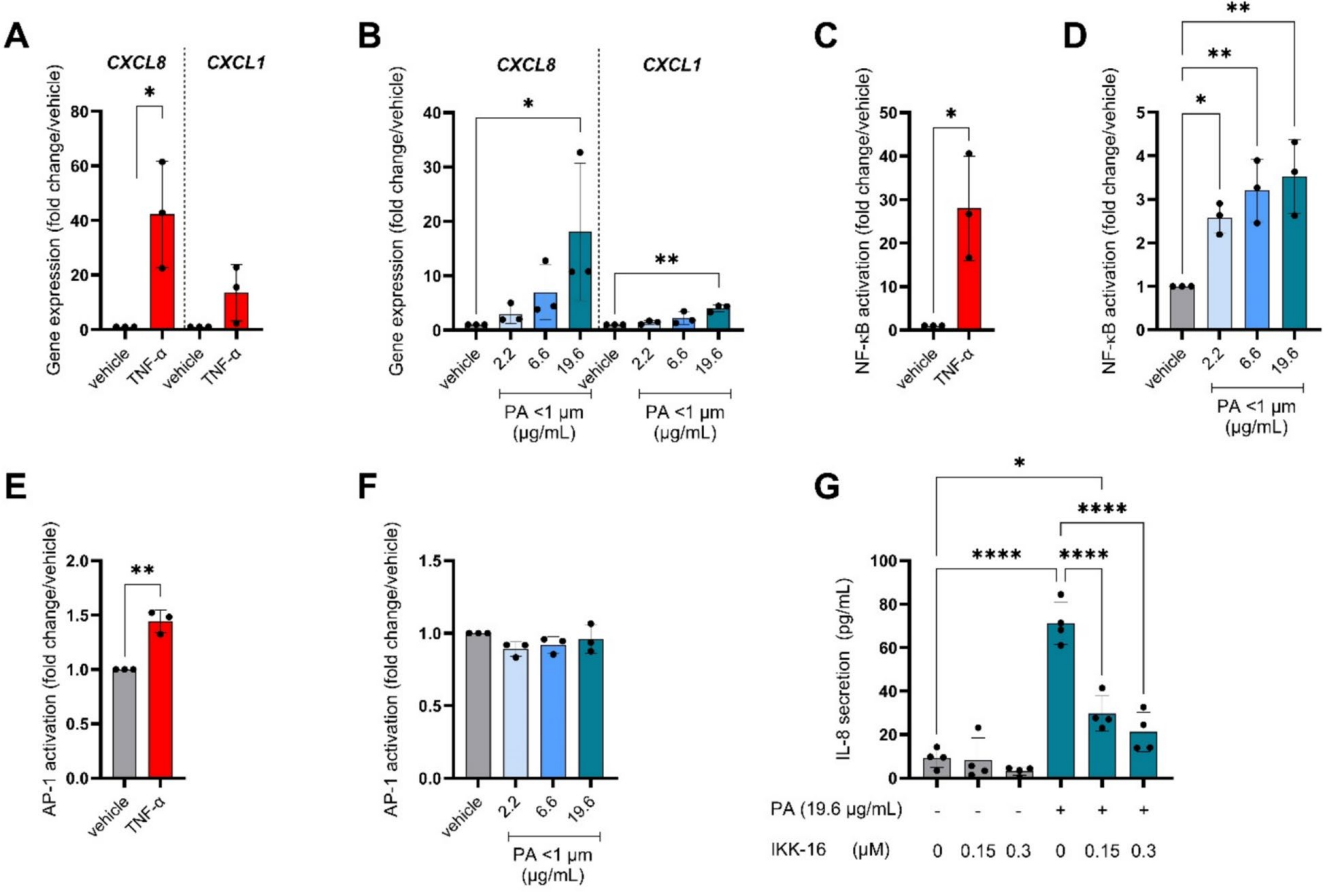
Pro-inflammatory gene expression and activation of NF-κB pathway upon exposure to polyamide (PA) nanoplastics. Cells were exposed to PA particles of < 1 µm (Applied doses are displayed in μg/mL), or TNF-α (50 ng/mL) in parallel experiments. **A**,** B** Gene expression of C-X-C Motif Chemokine Ligand 8 (*CXCL8*) and C-X-C Motif Chemokine Ligand 1 (*CXCL1*) **C**,** D** Nuclear factor kappa-light-chain-enhancer of activated B cells (NF-κB) activation was measured through luminescence intensity in stably transduced BEAS-2B cells with an NF-κB luciferase reporter. **E**,** F** Activator protein 1 (AP-1) activation was measured through luminescence intensity in stably transduced BEAS-2B cells with an AP-1 luciferase reporter. **G** IL-8 protein secretion after PA nanoplastics exposure, with different concentrations of NF- κB inhibitor (IKK-16). Every data point represents an independent exposure experiment, each performed in technical triplicate. Data are presented as mean fold change/vehicle control ± SD or absolute values (IL-8). Differences were considered statistically significant when *P* < 0.05. *P < 0.05; **P < 0.01; ***P < 0.001; ****P < 0.0001

To further investigate the underlying molecular mechanisms of IL-8 secretion induced by PA nanoplastics, we explored its impact on pro-inflammatory gene expression and inflammatory pathway activation. TNF-ɑ exposure, again as a positive control, increased expression of pro-inflammatory genes (Fig. 5A). In line with observations on IL-8 secretion, the PA nanoplastics induced *CXCL8* expression in a dose-dependent manner (Fig. 5B). Additionally, expression of *CXCL1* increased significantly but only in response to the highest tested dose (19.6 μg/mL/6.8 μg/cm^2^) (Fig. 5B). No significant changes were observed in expression levels of other pro-inflammatory genes (Fig. S4).

The *CXCL8* promoter contains binding sites for several transcription factors, including members of the NF-κB and AP-1 pathways. Therefore, we explored whether stimulation of the cells with PA nanoparticles induced the transcriptional activity of NF-κB or AP-1. As anticipated, TNF-α, known as a potent NF-κB activator, potently induced NF-κB transcriptional activity (Fig. 5C). NF-κB transcriptional activity was also significantly increased in response to PA compared to the vehicle control, in a dose-dependent manner (Fig. 5D**).** TNF-α also stimulated transcriptional activity of AP-1, although to a much lesser extent than NF-κB (Fig. 5E). In contrast to NF-κB activity, AP-1 transcriptional activity was not affected by PA exposure (Fig. 5F). Co-exposure with IKK inhibitor IKK-16 reduced NF-κB activity levels to vehicle-control levels without causing additional cytotoxicity (Fig S5). Inhibition of NF-κB significantly reduced PA-induced IL-8 secretion as well as *CXCL8* mRNA levels, in a dose dependent manner (Fig. 5G, Fig. S5B). These results show that PA-induced IL-8 release and inflammatory gene expression was indeed causally dependent on NF-κB activation.

### PA exposure did not induce intracellular ROS formation but upregulated *SOD2* antioxidant gene expression

**Fig. 6:**
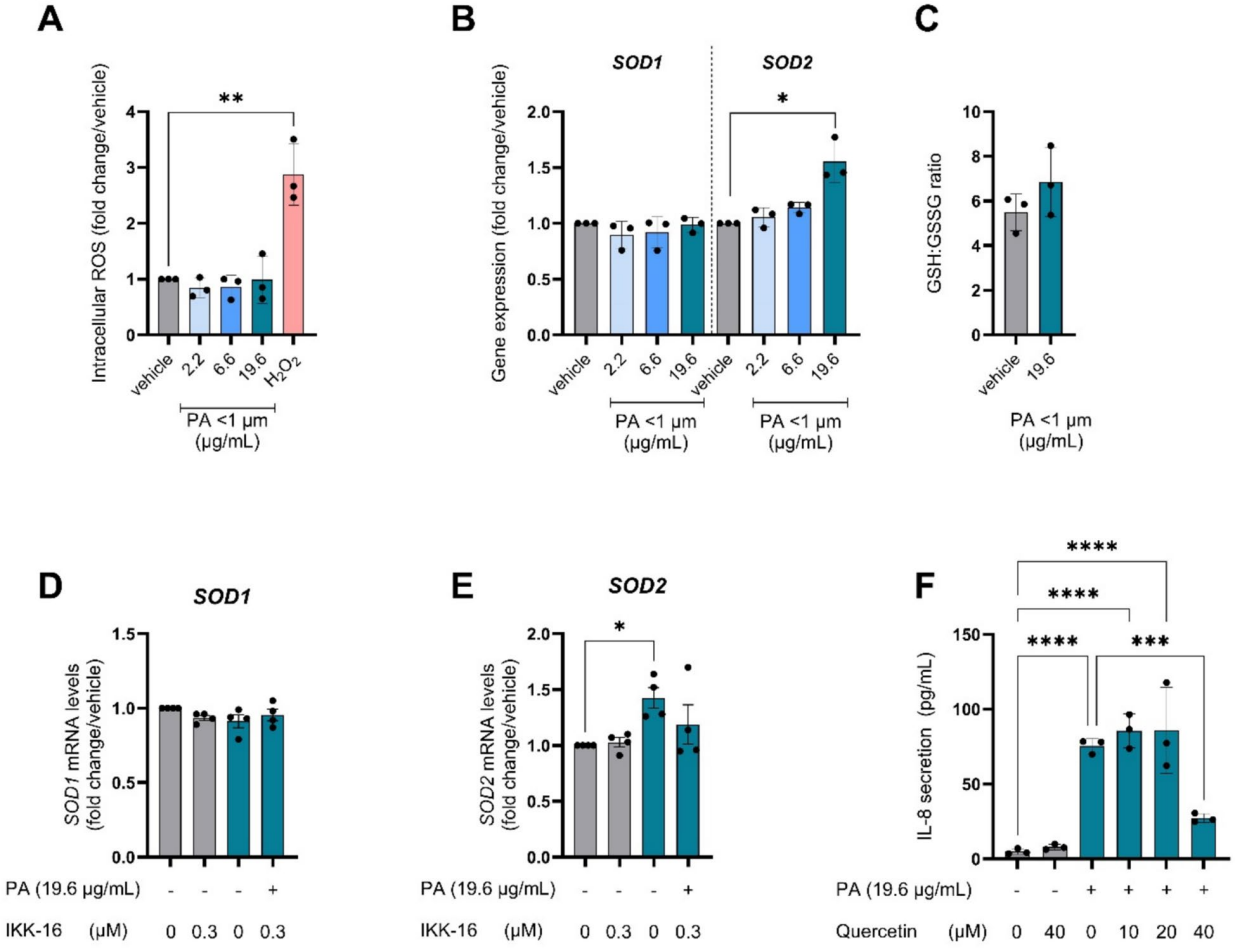
Antioxidant gene expression and intracellular ROS formation upon exposure to polyamide (PA) nanoplastics. Cells were exposed to PA nanoplastics (< 1 µm) (Applied doses are displayed in μg/mL) for 24 h. **A** Intracellular ROS was measured with the DCFH-DA assay **B** Gene expression of superoxide dismutase 1 (*SOD1*) and superoxide dismutase 2 (*SOD2*). **C** Ratio between reduced glutathione (GSH) and oxidized glutathione (GSSG). **D** Gene expression of *SOD1* after PA nanoplastics exposure, with different concentrations of NF-κB inhibitor (IKK-16) **E** Gene expression of *SOD2* after PA nanoplastics exposure, with different concentrations of IKK-16. **F** IL-8 protein secretion after PA nanoplastics exposure, with different concentrations of antioxidant quercetin. Every data-point represents an independent exposure experiment, each performed in technical triplicate. Data are displayed as mean fold change/vehicle control or absolute IL-8 protein levels ± SD. *P* < 0.05; *P < 0.05; **P < 0.01; ***P < 0.001; ****P < 0.0001

NF-κB can be activated in response to various stimuli, including ROS [30]. Firstly, in order to assess whether PA-induced toxicity was associated with modulation of cellular redox status, intracellular ROS as well as antioxidant gene expression was assessed after exposure of the cells to PA nanoplastics. Although H_2_O_2_ elicited a potent increase, intracellular ROS were not generated by PA treatment, as measured with the DCFH assay (Fig. 6A). With regard to cellular antioxidant gene expression, we observed that, while superoxide dismutase 1 (*SOD1*) gene expression was unchanged in response to PA, the expression of superoxide dismutase 2 (*SOD2*) was significantly increased after exposure to 19.6 µg/mL PA nanoplastics (Fig. 6B). Mitochondrial SOD2 can catalyze superoxide (O_2_^·−^) to H_2_O_2_, which subsequently is metabolized by the ROS-scavenging enzyme glutathione peroxidase (GPx) [31]. GPx, also one of the targets induced by NF-κB, requires the cofactor reduced glutathione (GSH), which is converted towards the oxidized glutathione (GSSG) [32]. We therefore measured the GSH:GSSG ratio. There was no significant change in the GSH:GSSG ratio after PA exposure (Fig. 6C).

Interestingly, PA-induced upregulation of *SOD2* gene expression was attenuated when NF-κB activity was inhibited (Fig. 6E), indicating that *SOD2* upregulation after PA exposure was NF-κB dependent. Inhibition of NF-ĸB did not affect *SOD1* mRNA expression (Fig. 6D). Moreover, after pre-incubation of cells with antioxidant quercetin, PA-induced IL-8 secretion was significantly reduced, showing involvement of ROS in the induction of NF-ĸB by PA (Fig. 6F). Collectively these two sets of experiments highlight the complex bidirectional interplay between NF-ĸB activation and modulation of cellular redox status in the context of PA-induced toxicity.

## Discussion

In contrast to spherical PS particles that are used by most studies addressing MNP toxicity, the MNPs in this study were derived from various polymers (PA, PVC, PP) and all displayed an amorphous shape. Using human bronchial epithelial cells, we observed that these environmentally relevant MNPs, in particular PA, induced a pro-inflammatory response and cytotoxicity in a concentration- and size-dependent manner. Also, PP/Talc MNPs exposure induced IL-8 secretion, however this could not be separated from IL-8 secretion induced by talc. PVC failed to induce these cellular responses at any of the doses and size fractions we investigated.

In line with our observations, previous studies have also demonstrated a size-dependent impact of MNPs on airway/pulmonary toxicity. For example, 80 nm PS nanoplastics caused more cytotoxicity in alveolar A549 epithelial cells than 2 µm PS particles [33]. A possible explanation for the observation that smaller MNPs elicit more, or more severe cellular toxicity responses compared to larger particles is that nanoplastics have a greater surface area relative to their mass [34]. Due to this increased surface area, there are more interaction sites available compared to particles with a small surface area, resulting in increased potential for the generation of cellular responses, more interactions with the epithelial cell membrane and higher levels of cellular uptake [35]. In addition, particle surface area, or particle number, has been considered to be a more appropriate dose metric than mass, in the field of particle toxicology [36]. When expressed as particle number, both the applied as well as the delivered dose was higher for the nanoplastics compared to the microplastics in our study, which may explain differences in cellular responses induced by the different size fractions.

In addition to a size-dependency in the severity of cellular toxicity elucidated by MNPs, we also observed differences in toxicity for MNPs originating from the different polymer types. It has to be mentioned that studies like ours, addressing toxicity of MNPs derived from environmentally-relevant polymers, are very scarce. This relates to the limited commercial availability of these MNPs which is likely linked to technical challenges associated with the production of larger amounts of smaller fractions of these polymers.

In our study, exposure to 19.6 µg/mL PVC MNPs, corresponding with ± 7 µg/cm^2^ deposition, did not lead to cytotoxicity. This finding is confirmed by another study in which cytotoxicity was absent when BEAS-2B cells were exposed for 24 h to 100 µg/mL 1 µm PVC MNPs [37]. However, in the same study, higher doses of PVC (200–800 µg/mL) decreased cell viability and resulted in upregulation of apoptotic genes as discovered through RNA sequencing [37]. Unfortunately, the authors did not report experimental parameters like exposure volume, therefore it was not possible to convert the applied dose (100—800 µg/mL) to an estimation of the deposited dose in µg/cm^2^ for direct comparison to our data.

Similar to PVC, PP/Talc exposure did not lead to cytotoxicity. However, PP/Talc, as well as talc alone, did induce IL-8 protein secretion in our study. Considering a low level of interference of PP/talc and IL-8 detection, the observed MNP-induced IL-8 secretion might be an underestimation of the actual cellular response [38]. The potential toxicity of PP in bronchial epithelial cells has not been studied so far. In alveolar epithelial A549 cells, PP nanoplastics induced mitochondrial dysfunction, cytotoxicity and NF-κB-regulated inflammation [39]. In contrast to the study on A549 cells, in our study talc was blended in the PP during its production to decrease buoyancy and increase particle deposition [22]. Talc, commercially considered as the most important filler for PP, improves its stiffness and stability [40]. However, the use of the talc has been subject to debate recently, and reclassified as carcinogenic by the European Chemicals Agency’s (ECHA) Committee for Risk Assessment (RAC) [41]. Despite a low particle deposition, Il-8 secretion was increased after nanotalc exposure. In agreement with our study, other studies also suggest increased cytotoxicity and inflammation in A549 cells exposed to nanotalc [42]. These effects were found to be size-dependent [42]. The high amount of agglomeration of the milled talc in exposure medium might explain why we see increased IL-8 secretion after exposure to non-milled talc but not to milled talc. The exact mechanism of talc carcinogenicity and the role of inflammation remains to be elucidated, since in vitro studies can only evaluate acute inflammatory effects.

The polymer type of MNPs we observed to have the strongest toxicity was PA. These findings correspond with the inflammatory response observed in rats, 24 h after intratracheal instillation of dust collected at a PA flocking plant [43]. Here, the authors observed an increase of macrophages and polymorphonuclear leukocytes in the lung upon PA exposure. In contrast, no pulmonary inflammation was reported upon both a four week (nose-only) in vivo exposure to PA fibers [44], nor an acute whole-body aerosol exposure to PA particles in rats [45]. In vitro studies regarding the effects of PA on cells of the airways and lungs are scarce, although a few studies exist. For example, A549 cells were exposed to PA nanoplastics for 72 h [46]. In contrast to our findings, doses up to 100 µg/mL (~ 62.5 µg/cm^2^) did not affect cell viability. Furthermore, a small increase in intracellular ROS levels was detected after 12 h, although this was only observed for the lowest dose tested (25 µg/mL ~ 16 µg/cm^2^). Noticeable factors that could contribute to discrepancies between the above-mentioned studies are cell-specific differences, exposure route and PA chemical subtype. The MOMENTUM PA particles have been tested in one other study, where the authors observed inhibiting effects of PA (1 μg/ml) and its leachate on the number of developing murine airway organoids [21]. However, this study did not investigate inflammation-related readouts.

IL-8 is known to be produced in response to various stimuli including pathogens, cigarette smoke and pollutants. Furthermore, IL-8 is relevant for chronic respiratory diseases as its expression in the airways and lungs is upregulated in diseases like COPD and Asthma [29]. The induction of IL-8 protein secretion and increases in inflammatory gene expression by PA nanoplastics that we observed was mediated by activation of the NF-κB signaling pathway. Although there is not much information on the molecular mechanism of PA-induced inflammation, several studies indicated NF-κB activation in BEAS-2B cells upon exposure to particulate matter [47], and nanoparticles including Zinc oxide [48]. Whether or not these effects and/or the effects of PA nanoplastics on NF-κB activation are mediated via interaction with certain surface receptors upstream of the NF-κB pathway or are mediated by intracellular events following internalization is unknown. A bidirectional link between NF-κB pathway activation and oxidative stress has been well-described in literature, although the exact interaction remains complex, since NF-κB can function both as anti- or pro-oxidant [30]. In this study, although we did not observe significant ROS or oxidation of GSH, we did detect increased antioxidant gene expression after PA exposure which was dependent on NF-κB activation. To the best of our knowledge this is the first report assessing redox status of bronchial epithelial cells exposed to PA. Also, use of an antioxidant (quercetin) attenuated PA-induced IL8 release indicating that ROS play a role in PA-induced activation of the NF-ĸB pathway. However, it has to be noted that the fact that we did not observe any changes in intracellular ROS levels in response to PA with the DCFH assay may be a result of particle interference with the assay, resulting in quenching of fluorescence emission [49]. Optimizations or alternatives for the DCFH assay need to be considered for future studies to accurately assess whether or not MNP exposure affects the level of intracellular oxidative stress [50].

A crucial aspect of interpreting in vitro MNP testing is accurately quantifying the dose that is delivered to target cells. In this study, we detected considerable variability in calculated deposition of all MNPs studied, influenced by their physicochemical properties and interaction with the exposure medium. This highlights the importance of incorporating not only the applied dose, but also the deposited dose in MNP in vitro hazard characterization**.** Furthermore, detailed particle characterization, as performed for the MNPs implemented in this study, is essential for reliable dose calculations and data interpretation [26, 51]. Corresponding with a previous study, we measured an absolute zeta potential < 30 mV for the MNPs in 1-propanol, indicating risk of agglomeration [52]. While in 1-propanol MNPs were close to their primary particle size, dispersion in exposure medium resulted in an increased size, especially for the smallest size fraction. These findings suggest binding of solvent molecules to the MNPs and agglomeration. Indeed, agglomeration was also observed during the exposure, especially when particles were applied in higher concentrations. Determining an appropriated environmentally-relevant in vitro dose is still challenging, due to the lack of optimal analytical techniques, especially for nanoplastics in the environment [53, 54]. Exposure through inhalation may vary substantially, since this is dependent on the amount of MNPs in the air, and individual inhalation rates. Median intake of microplastics (> 1 µm) through inhalation has been estimated to 1.07 x 10^-7^ mg/capita/day [1]. Daily airborne exposure has been estimated between 152–507 microplastics/kg body weight, based on environmental samples [54]. The same study estimated that maximal exposure levels in occupational settings were 22,531 microplastics/kg body weight during PP recycling processes. For a person of 88 kg, with a total airway surface area of 2471 ± 320 cm^2^, this would correspond with 710–922 particles/cm^2^ [55]. This number corresponds well with the deposited doses for PP/talc 5–10 µm microplastics (280–2500 particles/cm^2^). While the estimated real-life exposures to most MNPs correspond to lower doses than tested in our study, they do not account for accumulation of particles in the lung throughout lifetime, conferring higher concentrations, as well as longer exposure times. Moreover, deposition of MNPs in the respiratory tract is not uniform and highly depends on size and shape [56].

Some limitations need to be noted for this study. First of all, we have only examined acute exposure effects whereas we are continuously exposed to MNPs during a lifetime. Furthermore, the experiments in this study are performed on a cell line, grown under submerged conditions, which do not accurately reflect the physiological lung-environment that exists at the air–liquid interface. However, this cell system allowed us to screen all polymer types and sizes described in the paper in different doses, an endeavor that would have been much more challenging using animal work or more advanced in vitro systems. Exposure methods have been shown to affect both deposition and nanoparticle toxicity [57]. The milled amorphous MNPs used in this study are more environmentally relevant compared to commercially available MNPs, typically produced by precipitation of dissolved polymers. However, other environmental factors including UV-ageing and biodegradation are not taken into account in the present study. Since these factors can influence the physicochemical properties of MNPs and potentially cause additional adverse health effects, they should also be considered in future studies [58, 59].

## Conclusion

The present research is one of the first studies that comprehensively tests the toxicity of PVC, PP and PA particles in bronchial epithelial cells, and extends toxicity testing beyond spherically shaped PS particles. This study, as well as future research utilizing MNPs of a more diverse array of polymers could therefore more specifically inform public health regulatory bodies on risk assessment of MNPs.

## Supplementary Information

Supplementary Material 1

## Data Availability

The datasets used and/or analysed during the current study are available from the corresponding author on reasonable request.

## Abbreviations

MNPs: Microplastics and nanoplastics
PS: Polystyrene
ROS: Reactive oxygen species
PA: Polyamide
PET: Polyethylene terephthalate
PP: Polypropylene
PVC: Polyvinyl chloride
DMEM: Dulbecco’s Modified Eagle Medium
HBSS: Hank’s Balanced Salt Solution
dPBS: Dulbecco’s Phosphate-Buffered Saline
FBS: Fetal bovine serum
P/S: Penicillin–Streptomycin
TNF-α: Tumor necrosis factor-alpha
IL-8: Interleukin-8
MQ: MilliQ-water
SEM: Scanning electron microscopy
SE: Secondary electron
SLS: Static light scattering
V_pellet_: Pellet volume
ρ_media_: Media density
M_ENM_: Applied mass
ρ_ENM_: Bulk polymer density
SF: Stacking factor
ATCC: American Type Culture Condition
LDH: Lactate dehydrogenase
NEC: No enzyme control
NTC: No template control
*CXCL8*: C-X-C Motif Chemokine Ligand 8
*CXCL1*: C-X-C Motif Chemokine Ligand 1
*SOD1*: Superoxide dismutase 1
*SOD2*: Superoxide dismutase 2
*ACTB*: Beta-actin
*B2M*: Beta-2 microglobulin
*CYPA*: Cyclophilin A
*RPL13 A*: Ribosomal protein L13A
DCFH-DA: Dichlorofluorescein diacetate
KPE: Potassium phosphate lysis buffer
EDTA: EthyleneDiamineTetraaceticAcid
SSA: 5-Sulfosalicylic acid
GSSG: Glutathione disulfide
GSH: Glutathione
VP: 2-Vinylpyridine
NADPH: Nicotinamide adenine dinucleotide phosphate
DTNB: 5,5'-Dithiobis(2-nitrobenzoic acid)
IKK-16: Inhibitor of nuclear factor kappa B 16
NF-κB: Nuclear factor kappa-light-chain enhancer of activated B cells
AP-1: Activator protein-1
PSD: Particle size distributions
P.I.: Polydispersity index
D_N_: Median number-based size
D_V_: Median volume-based size
