# Supplementary material for: Size- and polymer-dependent toxicity of amorphous environmentally relevant micro- and nanoplastics in human bronchial epithelial cells
Source: Microplast nanoplast. 2025 May 16;5(1):19. doi: 10.1186/s43591-025-00126-9 (PMC12081513; doi:10.1186/s43591-025-00126-9)
Supplement: Supplementary file 1 — Supplementary Material 1 [file 43591_2025_126_MOESM1_ESM.pdf]

Supplemental Material

Size- and polymer-dependent toxicity of amorphous environmentally relevant micro- and nanoplastics in human bronchial epithelial cells

Gosselink IF[a#], Leonhardt P[a#], Höppener EM[b], Smelt R[a], Drittij MJ[a], Davigo M[a], van den Akker GGH[c], Kooter IM †[a,b], Welting TJM[c], van Schooten FJ[a], Remels AHV[a]

[a] Institute of Nutrition and Translational Research in Metabolism (NUTRIM), Department of Pharmacology and Toxicology, Maastricht University, 6229 ER Maastricht, the Netherlands

[b] Netherlands Organisation for Applied Scientific Research, TNO, 3584 CB, Utrecht, the Netherlands.

[c] Laboratory of Experimental Orthopedics, Department of Orthopedic Surgery, Maastricht University, 6229 ER Maastricht, the Netherlands.

[#] These authors contributed equally

Corresponding author:

Alexander H.V. Remels

Mail: a.remels@maastrichtuniversity.nl

Institute of Nutrition and Translational Research in Metabolism (NUTRIM)

Department of Pharmacology and Toxicology

Maastricht University Medical Center+

Universiteitssingel 50, 6629 ER Maastricht, the Netherlands

Table S1. Primer sequences used for real time quantitative PCR analysis

Gene	Sense primer (5'->3')	Antisense primer (3'->5')
ACTB	AAGCCACCCCACTTCTCTCTAA	AATGCTATCACCTCCCCTGTGT
B2M	CTGTGCTCGCGCTACTCTCTCTT	TGAGTAAACCTGAATCTTTGGA GTACGC
CYPA	CATCTGCACTGCCAAGACTGA	TTCATGCCTTCTTTCACTTTGC
RPL13A	CCTGGAGGAGAAGAGGAAAGA GA	TTGAGGACCTCTGTGTATTTGTC AA
CXCL8	TTAGAACTATTAAAACAGCCAA AACTCCACA	CAAGTTTCAACCAGCAAGAAAT TACTAATATTG
CXCL1	GAAAGCTTGCCTCAATCCTG	AACAGCCACCAGTGAGCTTC
IL1A	TTTCAGCCATCTTTGGAAGG	TCCTGAGCATTGACATCAGC
IL1B	GCACGATGCACCTGTACGAT	CACCAAGCTTTTTTGCTGTGAGT
MCP1	AGCAGCAAGTGTCCCAAAGAAG CT	CCTTGGCCACAATGGTCTTGAA
SOD1	GGTCCTCACTTTAATCCTCTAT	CATCTTTGTCAGCAGTCACATT
SOD2	TGGACAAACCTCAGCCCTAACG	TGATGGCTTCCAGCAACTCCC

Abbreviations: *ACTB*: beta-actin, *B2M*: beta-2 microglobulin, *CYPA*: Cyclophilin A, *Rpl13a*: ribosomal protein L13A, *CXCL8*: chemokine (C-X-C motif) ligand 8, *CXCL1*: chemokine (C-X-C motif) ligand 1, *IL1A*: Interleukin 1 alpha, *IL1B*: Interleukin 1 beta, *MCP1:* Monocyte Chemoattractant Protein-1, *SOD1*: Superoxide dismutase 1, *SOD2*: Superoxide dismutase 2

A

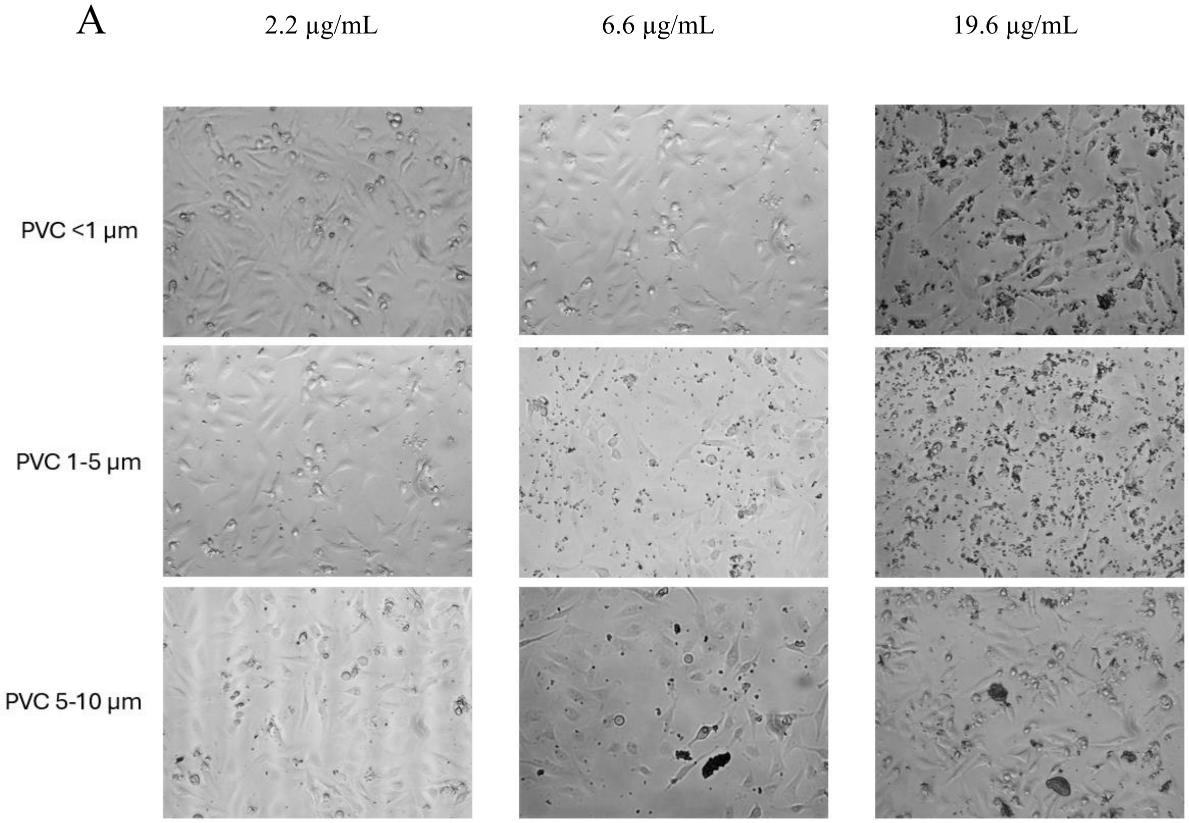
2.2 µg/mL
6.6 µg/mL
19.6 µg/mL
PVC <1 µm
PVC 1-5 µm
PVC 5-10 µm

B

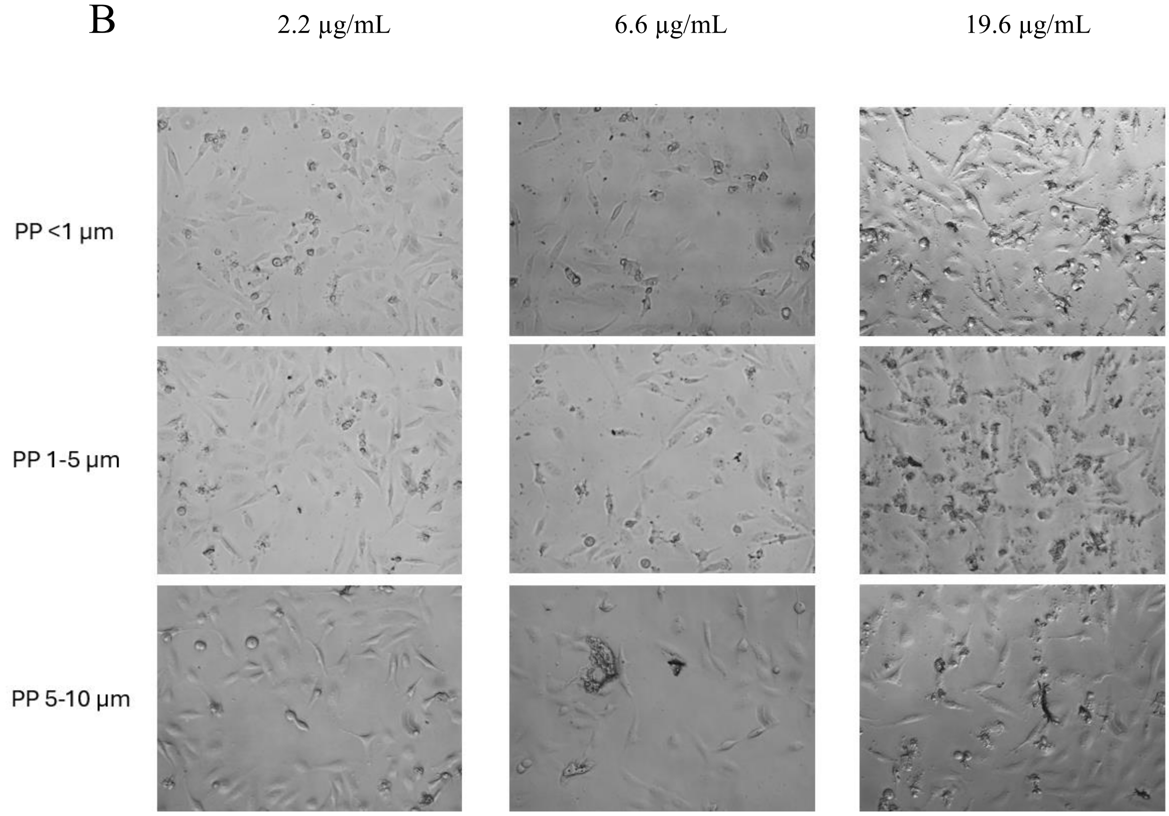
2.2 µg/mL
6.6 µg/mL
19.6 µg/mL
PP <1 µm
PP 1-5 µm
PP 5-10 µm

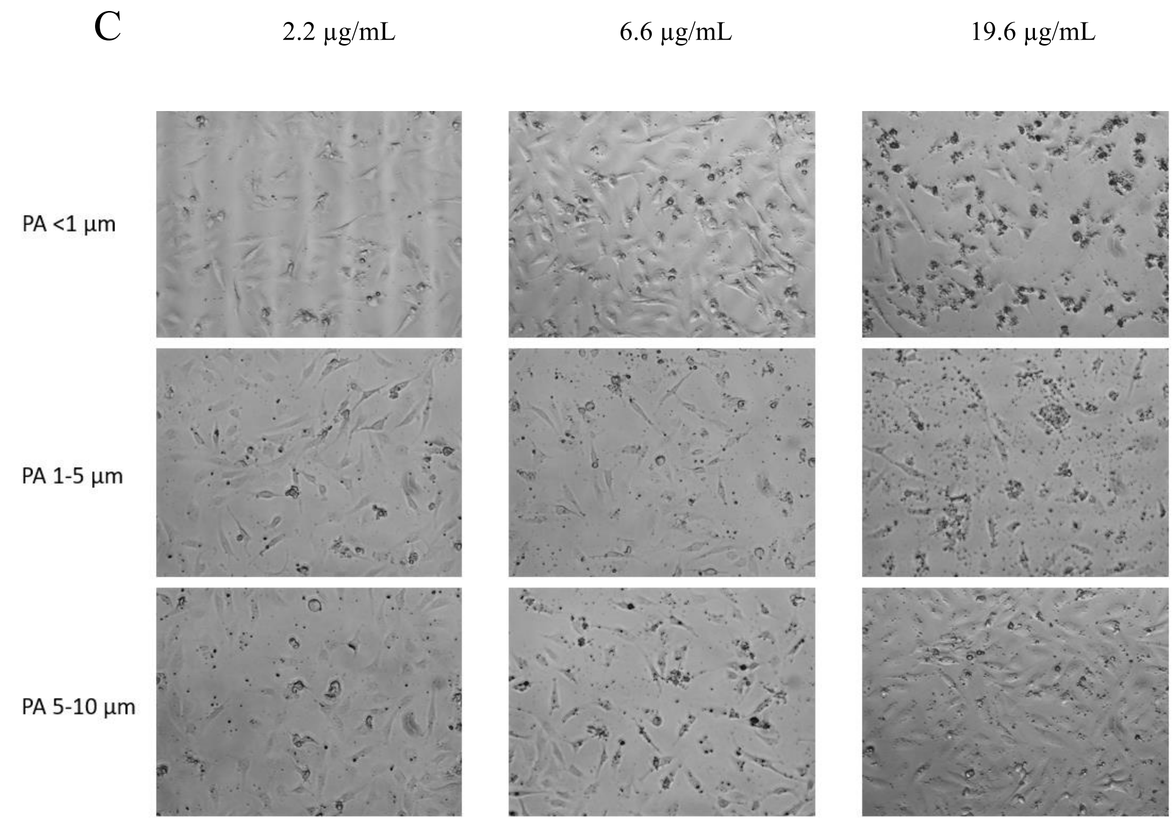

Fig. S1. Exposure of BEAS-2B cells to micro- and nanoplastics (MNPs). *Cells were exposed to particles of <1 µm, 1-5 µm or 5-10 µm at concentrations of 2.2 µg/mL, 6.6 µg/mL, or 19.6 µg/mL. Brightfield images of cells exposed for 24 h to* ***A*** *polyvinylchloride (PVC),* ***B*** *polyamide (PA) and* ***C*** *Polypropylene/talc (PP/Talc) MNPs.*

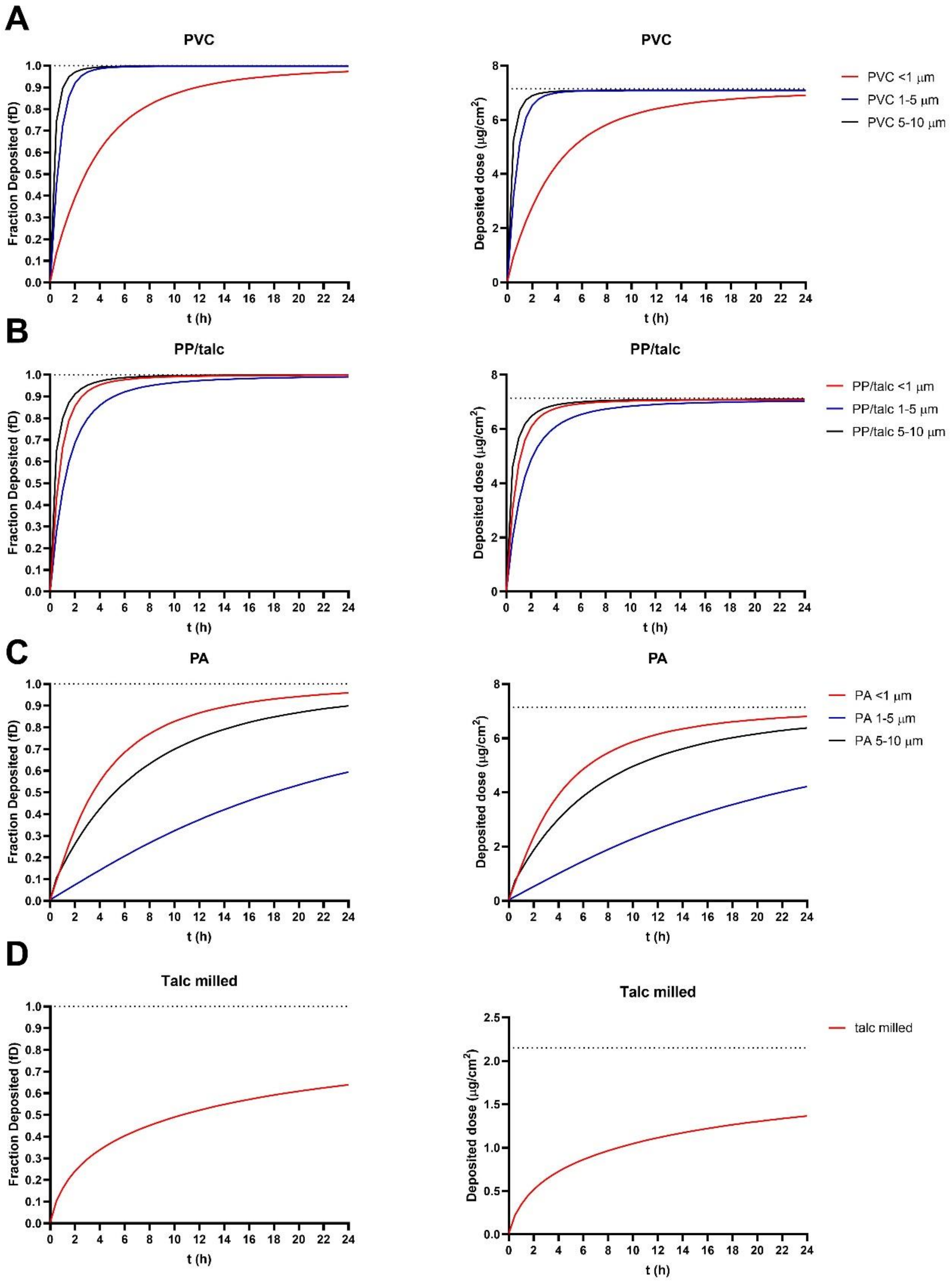

Fig. S2. Estimated deposited dose for micro- and nanoplastics (MNPs and talc). *Deposition was modelled voor each MNP over time, using the RiskGONE in vitro dosimetry web application. The dotted horizontal line represents 100% deposition of the applied dose for MNPs (19.6 µg/mL) or talc (5.9 µg/mL). The deposited fraction and deposited dose are displayed for* ***A*** *polyvinylchloride (PVC),* ***B*** *Polypropylene/Talc (PP/Talc) and C polyamide (PA) MNPs and* ***D*** *milled talc. Deposition of non-milled talc could not be estimated due to the low effective density.*

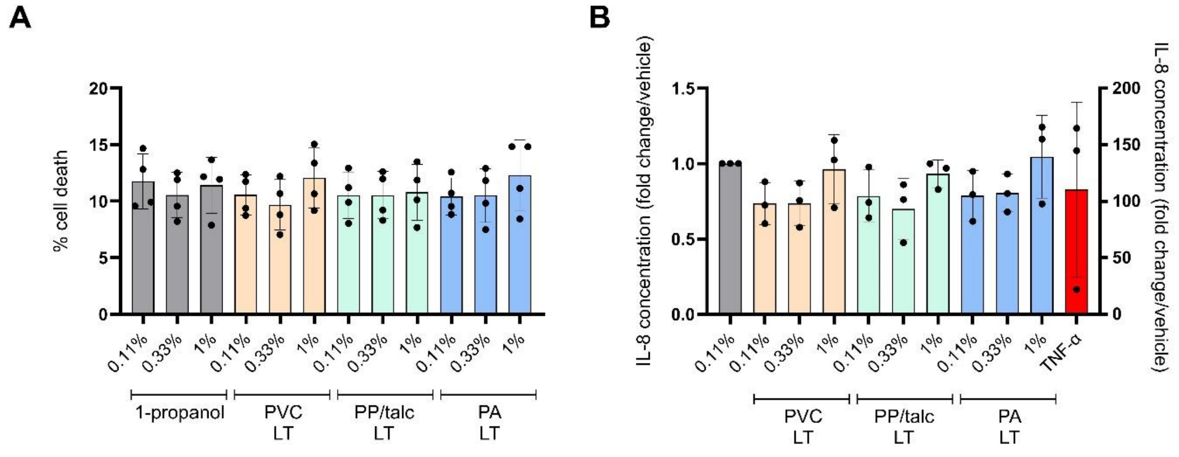

Fig. S3. Cytotoxicity and IL-8 secretion upon exposure to leachates (LTs) of polyvinylchloride (PVC), polypropylene (PP) and polyamide (PA) microplastics. *Cells were exposed to leachate solutions (0.11%, 0.33% and 1%) in exposure medium. Vehicle controls were adjusted accordingly (0.11%, 0.33%, and 1% of 1-propanol).* ***A.*** *Released lactate dehydrogenase (LDH) was measured after 24 h exposure. Maximum LDH release was achieved by exposure to Triton X-100 (2%).* ***B.*** *IL-8 concentration is presented as fold change over vehicle control. IL-8 secretion upon exposure to positive control TNF-α (50 ng/ml) or LTs. Pooled data of at least 3 independent exposure experiments, each performed in technical triplicate are presented as mean ± SD. ****P<0.0001 compared to vehicle control.*

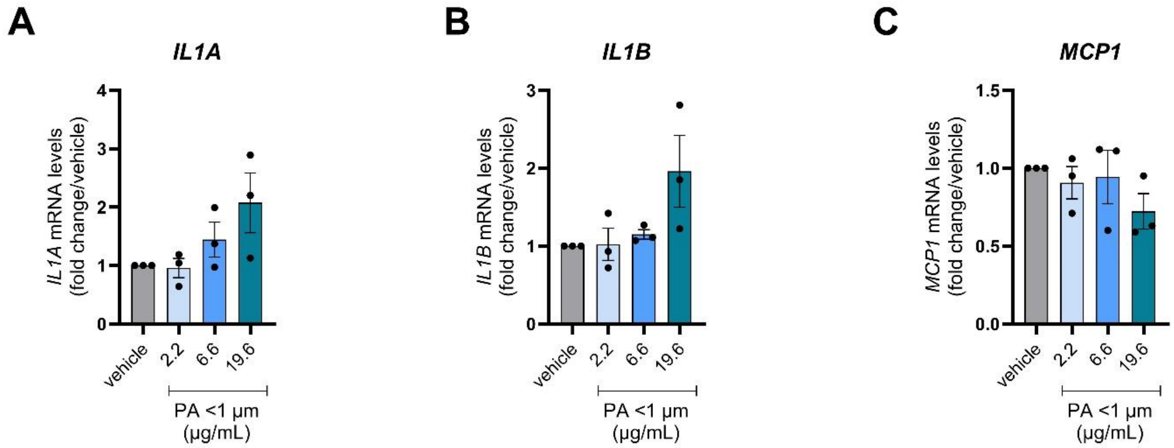

Fig. S4. **Pro-inflammatory gene expression upon exposure to polyamide (PA) nanoplastics.** *Gene expression of interleukin 1 alpha (IL1A)* ***(A)****, interleukin 1 beta (IL1B)* ***(B)*** *and Monocyte chemoattractant protein-1 (MCP-1/CCL2)* ***(C)***

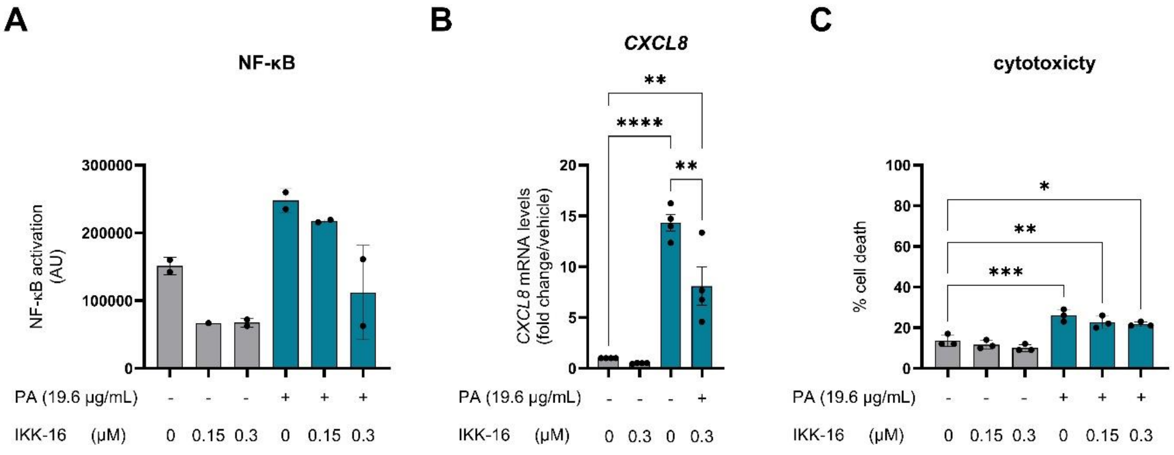

Fig. S5. The effect of NF- κB inhibitor (IKK-16) on PA-induced effects. *Cells were exposed to Polyamide nanoplastics in presence of different concentrations of Nuclear factor kappa-light-chain-enhancer of activated B cells (NF-κB) inhibitor (IKK16).* ***A.*** *NF-κB activation was measured through luminescence intensity in stably transduced BEAS-2B cells with an NF-κB luciferase reporter.* ***B.*** *Gene expression of C-X-C Motif Chemokine Ligand 8 (CXCL8)* ***C.*** *Cytotoxicity. Released lactate dehydrogenase (LDH) was measured after 24 h exposure. Maximum LDH release was achieved by exposure to Triton X-100 (2%).*

Table S2. Applied doses and estimated deposited doses for MNPs and Talc particles.

		Applied dose			Deposited dose					
		µg/mL			µg/cm²			# particles/cm²		
	Size fraction	Low	Medium	High	Low	Medium	High	Low	Medium	High
PVC	<1 µm	2.2	6.6	19.6	0.8	2.3	6.9	2.5E+06	7.4E+06	2.2E+07
	1-5 µm	2.2	6.6	19.6	0.8	2.4	7.1	5.4E+04	1.6E+05	4.8E+05
	5-10 µm	2.2	6.6	19.6	0.8	2.4	7.1	3.9E+03	1.2E+04	3.5E+04
PP/Talc	<1 µm	2.2	6.6	19.6	0.8	2.4	7.1	6.4E+05	1.9E+06	5.7E+06
	1-5 µm	2.2	6.6	19.6	0.8	2.4	7.1	7.4E+03	2.2E+04	6.6E+04
	5-10 µm	2.2	6.6	19.6	0.8	2.4	7.1	2.8E+02	8.3E+02	2.5E+03
PA	<1 µm	2.2	6.6	19.6	0.8	2.3	6.8	1.5E+05	4.6E+05	1.4E+06
	1-5 µm	2.2	6.6	19.6	0.5	1.4	4.2	6.7E+04	2.0E+05	5.9E+05
	5-10 µm	2.2	6.6	19.6	0.7	2.1	6.4	6.6E+04	2.0E+05	5.8E+05
Talc milled	<1 µm	0.7	2	5.9	0.2	0.5	1.4	6.5E+02	1.9E+03	5.5E+03
Talc non-milled	<1 µm	0.7	2	5.9	ND	ND	ND	ND	ND	ND

The deposited dose in µg/cm² is calculated based on the deposited fraction as calculated with the RISK gone in vitro model as well as the suspension column height (0.364 cm). The estimated number of particles per cm² is calculated from this mass-based dose using the following method:

First, we calculated the average volume per particle, based on the number-based particle size distribution of the stock (measured with SLS in 1-propanol).

$$V = \sum \frac{4}{3} \pi r_i^3 n_i$$

r_i = particle radius, n_i= fraction (%) of particles with radius i.

This number was used to calculate the number of particles per mL= 1/V

Lastly, we calculated the number of particles per gram microplastics by dividing the number of particles per mL (number of particles/cm³) by the polymer density (gcm⁻³). These calculations are based on the assumption of spherical particles. ND: For non-milled talc no deposition could be calculated, due to the low effective density of this particle in the exposure medium.
